# Maternal-Fetal Cancer Risk Assessment of Ochratoxin A during Pregnancy

**DOI:** 10.3390/toxins8040087

**Published:** 2016-03-23

**Authors:** Chit Shing Jackson Woo, Hani El-Nezami

**Affiliations:** School of Biological Sciences, Faculty of Science, Kadoorie Biological Sciences Building, The University of Hong Kong, Pokfulam, Hong Kong, China; jw86@connect.hku.hk

**Keywords:** Ochratoxin A, risk assessment, mycotoxin, fetal, pregnancy

## Abstract

Increasing evidence has demonstrated that *in utero* exposure to environmental chemicals may interfere with fetal development and increase the risk of disease and cancer development later in life. Ochratoxin A (OTA) has been proven to induce diverse toxic effects including teratogenicity, carcinogenicity, immunotoxicity and potential endocrine disruption. Due to the continuous and widespread occurrence of OTA as a potential contaminant of staple foods, there is increasing concern of *in utero* exposure of fetus to this mycotoxin. In this study, maternal-fetal risk assessment of OTA during pregnancy was conducted using the benchmark dose approach for genotoxic carcinogens. The daily intake of OTA for Egyptian pregnant women was estimated based on their serum OTA level using the refined Klaassen equation for pregnancy. Fetal exposure level was also estimated based on the maternal data. Comparison between the estimated daily exposure and the negligible cancer risk intake (NCRI), and the calculation of margin of exposure (MOE) implicated that OTA exposure from dietary intake would be of low health concern for this general subpopulation of Egyptian women. This subpopulation of pregnant women was generally estimated not to be in high-risk for toxicity induced by OTA.

## 1. Introduction

Mycotoxins and mycotoxicosis have been increasingly recognized as an important international issue over the last three decades because of their detrimental health effects and global occurrence [1]. Mycotoxins have been considered one of the most important chronic dietary risk factors [2]. Scientific and systematic risk assessment on their occurrence as food contaminants in different localities and exposure of populations, in particular the vulnerable subpopulation of pregnant women and their fetuses, is important. Recently, there has been increasing interest in developmental toxicology with growing awareness that a comprehensive risk assessment should also involve the assessment of *in utero* exposure and health effects [3].

Fetus has an exceptional vulnerability to detrimental health effects resulting from *in utero* exposure to environmental chemicals [4]. Gestation is a critical period for every species due to the involvement of important biological events such as organogenesis, developmental plasticity and endocrine programming. Exposure to toxic agents during gestation may interfere with the integrity of development and hence lead to adverse health outcomes later in life. With immature metabolism and poor detoxification system, fetal period probably is the most susceptible of all age groups to environmental stressors. Increasing evidence has also demonstrated that transplacental food-borne chemicals such as polycyclic aromatic hydrocarbons [5], flavonoids [6], aspartame [7] and aflatoxins [8] are able to cause or magnify adverse fetal health effects in animal studies and hence may also be harmful to human.

Ochratoxin A (OTA) is one of the mycotoxins of worldwide public health concern. Although risk assessment of OTA for the general public has been conducted and evaluated on a regular basis, the appropriate risk metric for OTA is still under debate due to the equivocal mechanism of OTA-induced carcinogenesis. Since its discovery, there have been no cases of human dietary intoxication being reported. In the absence of human OTA data, hazard identification and characterization mainly rely on extrapolation from experimental animal findings. With the advancement of analytical methodologies and the development of exposure biomarkers, human exposure to this food contaminant and its occurrence in food commodities have been reported worldwide. Although there is increasing concern about exposure of infants and children to environmental chemicals and risk assessment of OTA has been extended to infants and children in recent years [9,10], *in utero* exposure and fetal risk assessment have not been included. In light of the evidence regarding OTA-induced diverse health effects, transplacental transfer of OTA in laboratory animals [11,12,13,14,15], and higher level of OTA in human umbilical cord blood than maternal blood at the time of delivery [16,17], risk assessment of *in utero* OTA exposure is necessary [18]. In this study, risk assessment for Egyptian pregnant women and their fetuses was attempted.

## 2. Results

### 2.1. Hazard Characterization

#### 2.1.1. Derivation of the Benchmark Dose Lower Limit (BMDL_10_)

The total kidney tumor incidence in male rats from the National Toxicology Program (NTP) two-year rat study (Table 1) [19] was used as the cancer end-point of concern in deriving the BMDL_10_. Because the log-probit model had the lowest values for all the likelihood ratio, Akaike Information Criterion (AIC) and Goodness of Fit (GOF) tests, it fit the data adequately and hence was chosen as the model for BMDL derivation (Table 2). Derived BMDL_10_ using the log-probit method was adjusted from 22.5 to 16.1 μg OTA/kg bw/day (by multiplying the BMDL_10_ by 5 and then dividing by 7) due to the fact that male rats were dosed for only five days a week.

#### 2.1.2. Derivation of the Tumorigenic Dose (TD_05_) and Negligible Cancer Risk Intake (NCRI)

TD_05_ was also derived based on the total kidney tumor incidence in male rats from the NTP study (Table 1) [19] using the log-probit model. The derived TD_05_ was adjusted from 24 to 17 μg OTA/kg bw/day (by multiplying the TD_05_ by 5 and then dividing by 7) accounting for only five-days-a-week administration of OTA. Calculated NCRI based on the adjusted TD_05_ was 3.38 ng OTA/kg bw/day.

### 2.2. Exposure Assessment

In this study, 98 serum samples of pregnant women from Egypt were analyzed for OTA. OTA was detected in 80 samples with concentrations ranging from 0.20 to 1.53 ng/mL with mean value for positive samples with confidence interval at 95% and median as 0.33 ng/mL and 0.26 ng/mL respectively.

The estimated daily intake (EDI) based on the serum OTA level of the Egyptian pregnant women was calculated according to the refined Klaassen equation for pregnant women (Table 3). EDIs for pregnant women were calculated as 0.43 and 3.26 ng/kg bw/day for the low- and high-exposure groups, respectively, with a median at 0.55 ng/kg bw/day. Fetal EDI was assumed to be twice the maternal EDI (see Section 3.3.1 and Section 5.6.3). Fetal EDIs were estimated to be 0.86 ng/kg bw/day for the low-exposure group, 6.52 ng/kg bw/day for the high-exposure group and 1.10 ng/kg bw/day as the median.

### 2.3. Risk Characterization

#### 2.3.1. Pregnant Women

In order to provide more useful information for risk management, the Margin of Exposure (MOE) approach was used as a prioritization tool. Table 4 shows the MOE estimation and NCRI comparison. The estimated MOE (2.9 × 10^4^) based on the median exposure (0.55 ng/kg bw/day) for this subpopulation was generally higher than 1 × 10^4^ indicating low public health concern and low priority for risk reduction [20]. However, for the comparatively highly exposed individuals (3.26 ng/kg bw/day) in this study, the estimated MOE (4.9 × 10^3^) was just below 5000 suggesting a requirement for risk reduction. Similarly, comparing the derived NCRI (3.38 ng/kg bw/day) to the EDIs of OTA for pregnant women, the estimates of exposure were below the NCRI value (Table 3 and Table 4). The Egyptian pregnant women in general were estimated not to be under high risk.

#### 2.3.2. Fetus

With the evidence of fetal OTA exposure and diverse OTA-induced toxicities, special attention to this vulnerable subpopulation is warranted. However, available information on placental and fetal toxicokinetics throughout pregnancy as well as fetotoxicities of this mycotoxin is grossly lacking at the present time. Fetal risk assessment could only be conducted based on a very rough estimate of the fetal EDI. The fetal EDIs were below the NCRI value and the MOE values were higher than 1 × 10^4^ in the low- and median-exposure groups, suggesting potentially low health concern (Table 3 and Table 4). However, for the high-exposure group, the fetal EDI was almost twice the NCRI value and the MOE was 2.5 × 10^3^ suggesting a high priority for risk reduction.

## 3. Discussion

### 3.1. Hazard Characterization

Instead of the nephrotoxic endpoint obtained from the 90-day pig study [21,22,23], cancer endpoint obtained from the NTP two-year rat gavage study was selected to be a more appropriate point of departure (PoD) for hazard characterization using the non-threshold approach. As evidence by the high uncertainty factor (Table 5), the nephrotoxic data from the 90-day pig study have been considered inadequate for the estimation of the health-based exposure limit [9]. In addition, nephrotoxicity seems not to be appropriate as the endpoint in the chronic rat study reporting that kidney damage was neither a prominent factor nor causal factor for the renal carcinogenicity [9,24].

Although a similar risk assessment approach was used by Health Canada [9], different NCRI values were derived due to the adoption of different NCRI-estimate models. The multi-stage model in THRESH software was used by Health Canada while log-probit model in United States Environmental Protection Agency BMD software version 2.1.2.60 (EPA, Washington, DC, USA, 2010) was used in this study. The slight difference observed between NCRI derived by Health Canada (3.9 ng/kg bw/day) and current study (3.38 ng/kg bw/day) may be due to differences of software and model. This difference on model selection has reflected the existing concern on whether model selection should rely on statistics (how well the model fits the data), characteristics of the particular effect examined (threshold *vs.* non-threshold response), or numeric value (selection of the lowest BMD or BMDL). Currently, the average-model approach is suggested [27]. For the average-model approach, a weighted averaged BMD is computed based on the predicted responses and the relative goodness-of-fit of each model [27]. This average-model approach is considered to be a good approach because of the better characterization of uncertainty and precision. However, this new approach is still at the beginning stage. Validated executable software and guidance are not available at the moment.

### 3.2. Maternal Risk Assessment of OTA Based on Serum OTA Level

To date, most of the exposure estimations for risk assessment are based on consumption data of food commodities and corresponding levels of contaminants. The calculation requires data of the consumed amount of food commodities by individuals and OTA contamination level in respective food commodities. However, there is absence of national food consumption data for OTA probably because a total diet study would be very expensive and time-consuming. Therefore, alternatively it is more appropriate to monitor exposure biomarkers for exposure assessment because biomarkers reflect the actual internal dose and may provide more accurate and useful information regarding health effects attributable to the toxic substances. In the case of OTA, serum concentration could serve as an alternative for the calculation of EDI. However, the correlation between serum OTA concentration and OTA-contaminated food consumption is equivocal due to the inconsistent data among studies [28,29,30,31,32,33]. Calculation using the Klaassen equation may also underestimate the actual OTA intake as only renal clearance by glomerular filtration has been taken into account. Even in the refined Klaassen equation, a number of parameters relevant for pregnant women, such as placental clearance and the changes of physiological and biochemical parameters during pregnancy have not been taken into consideration. In the absence of actual experimental data regarding OTA toxicokinetics during pregnancy, the refined equation could not be validated. In this study, the maternal EDI could only be roughly estimated based on the maternal serum OTA level.

### 3.3. Fetal Risk Assessment of OTA from Maternal Exposure

Nowadays, estimates of fetal exposure to environmental chemicals mainly depend on the measurement of biomarkers of exposure from umbilical cord blood sample collected at delivery [34]. However, a single measurement at delivery does not accurately reflect the actual fetal exposure pattern throughout pregnancy and collection of fetal blood during gestation for scientific purposes would be difficult and ethically questionable. Estimating *in utero* exposure based on the maternal biomarkers of exposure or maternal dietary intake seems to be an appropriate approach, and feasible for fetal risk assessment. Particularly for *in utero* exposure assessment, monitoring maternal biomarkers of exposure, especially blood sample, could reflect the most relevant marker of bioavailability of the toxin in the maternal circulation. Theoretically, estimation of fetal OTA level from the maternal serum OTA level would be possible with the consideration of transplacental toxicokinetics at different gestational periods. However, information regarding gestational toxicokinetics such as placental clearance, relationship between maternal dietary intake and fetal serum level, and OTA-induced fetotoxicities as well as any potential subtle chronic effects on fetal growth and development is either insufficient or unavailable. Fetal OTA risk assessment based on a simple assumption of fetal serum OTA being twice the maternal serum OTA displays high degree of uncertainty, as is the case in the present study. The finding that fetal EDI was generally lower than the NCRI and that MOE estimate was above 1 × 10^4^, should therefore be interpreted with caution. There are two major considerations necessitating extra concern for fetal OTA risk assessment.

#### 3.3.1. Fetal Exposure to OTA during Early Pregnancy

Based on current available evidence, it is probable that transplacental transfer of OTA occurs since early gestational stage and accumulates in the fetal circulation towards the end of pregnancy. So far, two studies have compared the feto-maternal serum OTA level at delivery showing approximately double amount in the cord blood compared to maternal blood [16,17]. The higher bioavailability of OTA in the fetal circulation does not only confirm fetal OTA exposure, but also suggests possible active transport mechanisms of OTA from mother to fetus. Thinning of placental barrier towards delivery suggests that term placenta might be more permeable to environmental toxicants [35]. However, there is minimal transplacental transfer of OTA at late pregnancy demonstrated by human placental perfusion [36] and extensive transplacental OTA transfer only in early gestation in a rat study [37] supporting *in utero* OTA exposure beginning in early gestation. Estimation of *in utero* exposure throughout pregnancy based on the ratio of maternal-fetal serum OTA level at delivery may not accurately reflect the actual circumstance.

#### 3.3.2. Chronic and Early-Life Exposure Poses Extra Risk to the Individual

Human fetus is not a small adult. This vulnerable subpopulation is probably at a greater risk from carcinogenic environmental toxins because of high absorption rate, poor detoxification or elimination capacity, rapid cell proliferation, and immature repairing mechanisms. Increasing evidence has demonstrated that the fetus is more vulnerable to toxicant exposures than adults [4]. Human epidemiological studies have clearly shown that *in utero* exposure to environmental toxicants would contribute to the development of cancer early in life, or may even predispose to chronic disease and cancer later in life [3,38,39]. Elimination of toxic substances from our body mainly relies on two major processes: metabolism and excretion. Although fetal xenobiotic metabolism in the liver has been documented [40], most enzymatic processes are not well developed. Moreover, even if there is some excretion to fetal urine, this ends up in the amniotic fluid which is then subject to subsequent fetal swallowing [41]. Considering the potential endocrine disrupting and genotoxic properties of OTA, early exposure and continuous circulation of this mycotoxin in the fetus throughout pregnancy may interfere with critical fetal development. Early-life chronic exposure may increase susceptibility of an individual to diverse persistent health effects due to immature protective mechanism, disturbance in critical growth development, and longer latency for disease development.

### 3.4. Challenges and the Need of Fetal OTA Risk Assessment from Maternal Exposure

Inconsistency between human epidemiological findings [16,17] and *ex vivo* human placental perfusion [36] results raised the question about the transplacental kinetics of OTA at different gestational stages. Insufficient data regarding fetal exposure to OTA and its toxicokinetics during pregnancy leads to a great challenge for exposure assessment. Moreover, although there is a growing research interest in *in utero* exposure and related health effects on childhood and adulthood, information for fetal risk assessment is insufficient. Changes in physiology and biochemistry during pregnancy, gestational pharmacokinetics of the substance, and fetal susceptibility are the important factors, which require extra consideration in fetal risk assessment process. Continuous effort is required to provide comprehensive information about the relationship between dietary exposure data and maternal or even fetal internal dose level of the toxin at different gestational stages. This is important due to the potential long-term effects of early-life exposure on individual health.

Data from fetal and childhood risk assessment should also be considered as part of the general risk assessment of OTA. OTA is one of the 20 mycotoxins known to occur globally in foodstuffs at significant levels and frequencies to cause public health concern [42]. High incidence of OTA exposure reported in epidemiological studies from more than 20 countries suggested that the population ranging from the fetus to the elderly has been and is being exposed to OTA in a continuous and widespread manner [29,43,44,45,46]. Human fetuses are exposed to OTA through maternal exposure, newborns are exposed through OTA-contaminated human breast milk or infant formula, children and adults are exposed through OTA-contaminated food and derived products. Considering OTA as a chronic dietary risk factor, a more conservative approach is suggested for the risk assessment of OTA especially for the fetus because an adaptive response during fetal development to *in utero* exposures could result in persistent influence on health later in life [38].

## 4. Conclusions

Non-threshold approach based on cancer endpoint was adopted in this study for OTA risk assessment because of the potential genotoxic property. Based on the comparison with the NCRI and the estimated MOE, both Egyptian pregnant women and their fetuses in general were not under high risk for OTA-induced toxicity except for the high-exposure group. However, due to limited information about *in utero* exposure and the fetotoxicity of OTA, a high degree of uncertainty remains in the estimated fetal exposure to this mycotoxin during gestation and consequently in the possibility to OTA-induced fetotoxicity. Fetal risk associated with maternal exposure could only be estimated. Considering the sensitivity of fetus to toxicity, a high priority for risk reduction, especially reduction of exposure if high-exposure is suspected would be important. Generally, keeping exposure to OTA at a minimum in Egyptian women, in particular during pregnancy, is recommended.

## 5. Materials and Methods

### 5.1. Chemicals

Ochratoxin A standard was purchased from Supelco Analytical (Bellefonte, PA, USA). Gradient grade acetonitrile and methanol were purchased from Merck (Darmstad, Germany) and glacial acetic acid from BHD Chemicals (Poole, UK). Strata C18-E solid phase extraction columns (50 mg, 1 mL) were purchased from Phenomenex (Torrance, CA, USA).

### 5.2. Sample Collection

Study participants were recruited during May–September 2006 from Elmenofya governorate, Egypt, where exposure to dietary mycotoxins is in widespread manner due to the environmental conditions. The Elmenofya governorate is located in the eastern Nile region, close to the head of the Delta. Most of the population in this governorate lives in rural areas with income from agriculture. Ninety-eight pregnant women in their last trimester were informed about this study and gave their written consent for their participation. The study was approved by the Ethical Committee of Ministry of Health in Egypt (1664, 11 October 2005). To determine OTA exposure in the studied population, serum samples were collected and kept at −20 °C until analyzed. Individual food frequency questionnaires including milk products (dairy milk, milk powder, and cheese), corn (dried corn, grilled corn, and corn flour), legumes (beans, lentils, and chick peas), fruits (fresh figs, dried figs, and apples), oils (cotton seed, sesame, corn, olive, sunflower, and margarine), and spices and nuts (peanuts, pistachios, and nuts) were obtained. Socio-economic data (working status, income, and level of education), demographic data (place of residence, and age) and clinical data (health status and medication) were also recorded by interview. As this study was the extended part of a mycotoxin intervention study, the questionnaire was established to capture aflatoxin sources, but not OTA sources, which would include wheat. All samples were assayed with the laboratory personnel being blinded to the subject information.

### 5.3. Extraction of Serum OTA

Serum OTA was extracted by solid-phase extraction (SPE) based on Ghali *et al.* [47] with some modifications. 0.5 mL of serum sample was acidified with 15 μL of acetic acid. The acidified serum was then passed through the SPE-cartridge (Stata-C18E, 50 mg, 1 mL) previously conditioned with 6 mL of absolute methanol under vacuum. The cartridge was washed with 4 mL of distilled water and the bound OTA was eluted with 6 mL of acidified methanol (methanol/acetic acid, 95/5). The eluate was evaporated to dryness and reconstituted with 0.5 mL of absolute methanol prior to HPLC analysis.

### 5.4. Analysis of OTA by High Performance Liquid Chromatography

The HPLC equipment consisted of a WATERS HPLC system (Waters, Milford, MA, USA) including a separation module (Waters 2695) equipped with Waters Symmetry C18 column (4.6 × 250, 5 µm) kept at +30 °C. The mobile phase consisted of acetonitrile:water:acetic acid (50/49/1) with flow rate of 1 mL/min. Fluorescence detection was done using 310 nm as excitation wavelength and 465 nm as emission wavelength using a WATERS model 2475 fluorescence detector. For OTA quantification, a 7-point calibration curve (0.2–10 ng/mL) covering the range of interest for the test samples was used. The OTA levels were quantified by interpolation in the calibration curve. All standards and samples were analyzed as duplicates with injection volume of 10 µL. The limit of detection (LOD) for OTA was set to 0.2 ng/mL based on a signal/noise of 3:1 after repeated testing. OTA identity in all samples was confirmed by spiking the samples with OTA standard.

### 5.5. Data Management and Statistical Treatment of the Data

Data management and statistical analysis were performed with the SPSS 16.0 Microsoft version (SPSS Inc., Chicago, IL, USA, 2007). For statistical analysis all samples below LOD were assigned as half of this value. Mean and median of positive samples were determined.

### 5.6. Risk Assessment

Risk assessment, in the field of food safety, is an integral scientific process to assess the probability of known or potential adverse health effects in human after the exposure to toxic agent in food. Risk assessment process consists of four major components including hazard identification, hazard characterization, exposure assessment, and risk characterization.

#### 5.6.1. Hazard Identification

Hazard identification has been defined as ”the identification of the type and nature of adverse effects that an agent has an inherent capacity to cause in an organism, system, or (sub)population” [48].

OTA is the most potent renal carcinogen known to date [49]. It has also been implicated as a potential factor in the etiology of urinary tract tumor (UTT) associated with Balkan Endemic Nephropathy (BEN) [50,51,52]. In addition, OTA has been suspected as a cause of testicular cancer based on ecological analysis between the incidence of testicular cancer and the principal dietary sources of OTA in 20 countries [53]. Recently, the possible role for OTA in testicular cancer has been further supported by the detection of testicular DNA adduct in mice offspring after *in utero* exposure [54]. However, molecular events of OTA-induced carcinogenesis are still a hotly debated topic. Both non-threshold and threshold mechanisms have been hypothesized in OTA-induced carcinogenesis. Negative results from most of the conventional mutagenicity assays and inconsistent findings from DNA adduct studies have been reported. Findings in favor of a threshold mechanism have suggested the interacting involvement of modulation of the activities of transcription factors, alterations of cell signaling pathway, inhibition of protein synthesis, and generation of oxidative stress (for review see [55,56,57]).

On the other hand, OTA is suggested to be bioactivated to electrophilic species which bind covalently to DNA resulting in DNA adduct formation [58,59]. The para-chlorophenolic moiety of OTA undergoes oxidative dechlorination to form a quinone species [60,61] which has been suggested to be involved in covalent modification of DNA [59,62]. It has been proposed that the marked gender difference associated with OTA-induced carcinogenicity in rats could result from higher expression of activating enzymes in males in line with its known biotransformation to toxic metabolites [19,59]. Although conflicting results regarding OTA-DNA adduction have been obtained (for a review see [63]), recent structural determination of the principal adduct demonstrated direct covalent binding of OTA to DNA [64] which provided evidence about the genotoxic potential of OTA. In addition, the characteristics of OTA-induced tumors in the NTP study are similar to those typical for genotoxic carcinogens (Table 6) [9]. However, because of the complex biological events and the poor bioactivation of OTA into DNA reactive species, there have been divergent opinions of the interpretation of the mode of action (MOA) of OTA-induced carcinogenesis for decades. Currently, there is no international consensus among various regulatory and advisory bodies about the potential carcinogenic risk of OTA.

It is clear that OTA is a carcinogen in rodent, in particular to kidney [49], and poultry [65] but inconsistent evidence has been provided as to its genotoxic properties [59,64,66,67,68,69,70,71,72]. The existence or predominance of threshold carcinogenesis cannot be sufficiently supported. According to the classification of chemical carcinogens proposed by Bolt *et al.* [73], OTA should be classified as a borderline case with insufficient support for threshold genotoxicity. Based on the equivocal genotoxic status of OTA, linear non-threshold extrapolation was used as the default assumption, which should be prudent for the protection of public health. Being classified as a substance with carcinogenic and potential genotoxic properties, non-threshold approach was regarded feasible for OTA risk assessment.

#### 5.6.2. Hazard Characterization

Hazard characterization has been defined as “the qualitative and, wherever possible, quantitative description of the inherent property of an agent or situation having the potential to cause adverse effects” [48].

In this study, benchmark dose lower limit (BMDL) was used as the point of departure (PoD) for margin of exposure (MOE) calculation. BMDL referring to the corresponding lower limit of a one-sided 95% confidence interval on benchmark dose (BMD) was calculated using the United States Environmental Protection Agency BMD software version 2.1.2.60 (EPA, Washington, DC, USA, 2010). Although statistically a 50% extra risk is considered as the best PoD in MOE calculation, it may not take into account the shape of the dose-response relationship and modeling of lower values for the BMD approach such as 1% or 5% may also introduce greater uncertainty [27]. On basis of this, 10% risk level for benchmark response (BMR) regarding cancer endpoints as recommended by the International Life Sciences Institute (ILSI) Europe Risk Assessment of Genotoxic Carcinogens in Food Task Force [27] was adopted in this study. Dichotomous modeling was used as a BMD model for the derivation of BMDL_10_ based on the total kidney tumor incidence in male rats from the NTP study (Table 1). Likelihood ratio test, Akaike Information Criterion (AIC) and Pearson chi-square “goodness-of-fit” (GOF) test were implemented to validate the degree of model fit.

In addition to the MOE approach, negligible cancer risk intake (NCRI), which is health-based exposure metric for genotoxic carcinogens, suggested by Health Canada was also calculated and compared with the estimated daily intake (EDI) [26]. Tumorigenic dose 05 (TD_05_) was derived by the same model of BMDL. NCRI was calculated by dividing the TD_05_ by 5000, which is equivalent to linear extrapolation to zero exposure. NCRI provides similar estimation of “safe” intake to the tolerable daily intake (TDI) derived from low dose linear models, and it is suitable for non-threshold carcinogens and even threshold carcinogens with uncertain MOA [26].

#### 5.6.3. Exposure Assessment

Exposure assessment has been defined as “the evaluation of the exposure of an organism, system, or (sub)population to an agent (and its derivatives)” [48]. Human dietary exposure estimated from food consumption is the most common method for exposure assessment of food related substances. Apart from this, exposure can also be estimated based on measurements of exposure biomarkers and pharmacokinetic relationships. Because exposure to dietary mycotoxins is common in Egypt due to environmental conditions [77,78,79,80], this country was chosen as a model for risk assessment in this study.

Due to insufficient information concerning OTA contamination levels in different food commodities and food consumption data of pregnant women in particular, exposure assessment based on the measurement of Egyptian maternal serum OTA levels was adopted. EDI was calculated according to the refined Klaassen equation for pregnant women. Fetal exposure level was also estimated based on the maternal serum OTA level from this project.

##### Pregnant Women

As an alternative to food consumption data, daily intake of OTA was estimated from its plasma concentration based on pharmacokinetic relationships. Klaassen question [31] relating continuous dietary intake of OTA (*k*_0_, ng·kg^−1^ body weight per day) with plasma concentration (*C*_p_, ng·mL^−1^), renal clearance (*Cl*_renal_, mL·kg^−1^ body weight per day) and bioavailability (*A*, the fraction of OTA absorbed from intestine) was applied to estimate daily intake.

*k*_0_ = *Cl*_renal_ × *C*_p_/*A* (1)

In general, renal clearance of 0.67 derived from the clearance of insulin [81] (as cited in [31]) or 0.99 based on a single human experiment on OTA kinetics are commonly used [82] (as cited in [31]). Bioavailability of OTA is estimated to be 0.5 (50%). However, changes in the cardiovascular system, respiratory system, composition of body fluids, renal function, activity of hepatic enzymes, and the gastrointestinal system during pregnancy can potentially lead to alterations in the toxicokinetic processes of absorption, distribution, and elimination [41,83]. Estimation of OTA intake for pregnant women through OTA plasma level was refined. 

During pregnancy, the relaxing effect of elevated progesterone on smooth muscle is suggested to be responsible for the reduction in intestinal motility leading to 30%–50% increase of gastric emptying and gastrointestinal transit time [84,85,86,87]. Because OTA is mainly absorbed in the stomach and small intestine, longer retention time in the gastrointestinal tract potentially increase the absorption of OTA leading to higher bioavailability. Therefore, 0.7 (0.5 × 140%) instead of 0.5 for the bioavailability value was used.

Changes in effective renal plasma flow, glomerular filtration rate and endogenous creatinine clearance are among the most significant physiological changes in normal pregnancy and most probably affect OTA kinetics since OTA is excreted from the body predominantly by the urinary pathway. It has been reported that drugs which are excreted primarily unchanged in the urine display enhanced elimination and lower steady state serum concentrations during pregnancy because of a 50% increase in renal plasma flow and glomerular filtration rate during pregnancy [88,89]. Renal clearance was increased from 0.99 to 1.49 (0.99 × 150%) based on the OTA renal clearance value obtained from the single human experiment. On basis of theses considerations, we refined the Klaassen equation for pregnant women as below:

*k*_0_ = 1.49 × *C*_p_/0.7 = 2.13 × *C*_p_ (2)

##### Fetus

With limited information regarding the feto-maternal serum OTA levels during pregnancy, estimated fetal serum level of OTA was based on the two-fold difference between maternal and fetal serum at delivery reported by Zimmerli [16] and Postupolski [17]. Based on this we assume the EDI for fetus to be twice the maternal EDI.

#### 5.6.4. Risk Characterization

Risk characterization has been defined as “the qualitative and, wherever possible, quantitative determination, including attendant uncertainties, of the probability of the occurrence of known and potential adverse effects of an agent in a given organism, system, or (sub)population, under defined exposure conditions” [48]. It usually involves the comparison and derivation of a ratio between daily exposure over a lifetime dose and the health-based exposure limit under which the risk is considered as insignificant. MOE has been recommended by European Food Safety Authority (EFSA) as the harmonized approach for risk assessment of substances which are both genotoxic and carcinogenic [20].

In this study, MOE with respect to the cancer endpoint was calculated by dividing BMDL_10_ by the EDI for Egyptian pregnant women. In supplement to the MOE approach, NCRI was also compared with the EDI. With limited information regarding *in utero* exposure to OTA and relevant toxicological information, fetal risk associated with maternal exposure during pregnancy could only be inferred based on the maternal data.

## Figures and Tables

**Table 1:** Data of renal tubular cell tumors in male rats in the two-year gavage study [19].

Dose (μg OTA/kg bw/Day)	Number of Animals in the Dose Group	Number of Animals with Renal Carcinomas	Number of Animals with Renal Adenomas	Number of Animals with Both Renal Carcinomas and Adenomas
0	50	0	1 (2%)	1 (2%)
21	51	0	1 (2%)	1 (2%)
70	51	16 (31%)	6 (12%)	20 (39%)
210	50	30 (60%)	10 (20%)	36 (72%)

**Table 2:** Calculation of Benchmark Dose Limit (BMD_10_) and Benchmark Dose Lower Limit (BMDL_10_) using dichotomous stochastic Benchmark Dose (BMD) models based on total kidney tumor incidences in male rats from the National Toxicology Program (NTP) study [19] ^a^.

Model	Number of Parameters	Likelihood Ratio Test		Akaike Information Criterion (AIC)	Goodness of Fit (GOF) Test		BMD_10_ (μg/kg bw/Day)	BMDL_10_ (μg/kg bw/Day)
		Log-Likelihood	*p*-Value		χ^2^	*p*-Value		
Null	1	−121.11	-	-	-	-	-	-
Logistic	2	−82.22	<0.01	168.4	16.33	<0.01	52.4	42.9
Probit	2	−81.32	<0.01	166.6	15.02	<0.01	49.2	40.8
Quantal-linear	2	−77.88	0.01	159.8	6.14	0.05	18.6	14.8
Gamma multi-hit	3	−76.43	0.02	158.9	5.01	0.03	31.0	18.2
Multistage	3	−77.39	0.01	160.8	6.10	0.01	24.4	15.5
LogLogistic	3	−75.64	0.05	157.3	3.55	0.06	31.9	20.7
LogProbit	3	−75.10	0.09	156.2	2.70	0.10	33.2	22.5
Weibull	3	−76.76	0.01	159.5	5.38	0.02	28.7	16.9
Full	4	−73.63	-	-	-	-	-	-

^a^ Ochratoxin A administered by gavage 5 days/week for two years.

**Table 3:** Estimated Maternal and fetal daily intakes.

Exposure	High	Median	Low
Pregnant women			
Serum Level	1.53 ng/mL	0.26 ng/mL	0.20 ng/mL
Estimated Daily Intake (EDI)	3.26 ng/kg bw/day	0.55 ng/kg bw/day	0.43 ng/kg bw/day
Fetus			
Serum Level	3.06 ng/mL	0.52 ng/mL	0.40 ng/mL
EDI	6.52 ng/kg bw/day	1.10 ng/kg bw/day	0.86 ng/kg bw/day

Note: Fetus values were estimated based on maternal values.

**Table 4:** Estimation of Margin of Exposure (MOE) and Negligible Cancer Risk Intake (NCRI) comparison.

Exposure	High	Median	Low
Pregnant women			
MOE	4.9 × 10^3^	2.9 × 10^4^	3.7 × 10^4^
NCRI comparison	<NCRI	<NCRI	<NCRI
Fetus			
MOE	2.5 × 10^3^	1.5 × 10^4^	1.9 × 10^4^
NCRI comparison	>NCRI	<NCRI	<NCRI

**Table 5:** Recommended uncertainty factor for Tolerable Daily Intake (TDI) method using data from 90-day pigs study.

Uncertainty Factor	Reason (References)
10	Intraspecies difference [25].
25	Interspecies difference based on half-life difference between pigs and human with the same route of exposure (oral) [9].
10	Lowest Observed Adverse Effect Level (LOAEL) → No Observed Adverse Effect Level (NOAEL)
	In the absence of NOAEL, UF of 3 should be applied if the LOAEL is of sufficient quality. Because of the small number of animals per group, a more conservative UF up to 10 is reasonably applied [25].
1–10	Highly susceptible life-stages such as childhood and pregnancy [26].
>2500	If all of the above taken on account.

**Table 6:** Characteristics of animal tumors induced by threshold and non-threshold carcinogens [9,74].

Characteristics	Threshold	Non-Threshold	OTA	References
Species	Often only in single species	Not restricted to single species	Shown in two species	[19,75]
Sex	Single	Both	Both sexes	[19]
Site	Single	Multiple	Liver and kidney (both sex) Breast (female).	[19]
Tumorigenic potency	Low	High	Unusual high incidence of renal cell carcinomas (60%) ^a^	[49,76]
Ratio of carcinomas to adenomas	Low	High	High	[19]
Aggressiveness	Mutation frequency similar to spontaneous tumors	Rapid progression	High degree of atypia, rapid progression, large size (2–6.5 cm) ^b^, and invasive	[19,76]
		Often bilateral and multiple		
		High cytoplasmic atypia; invasive		
Metastases	Rare	More common	Common ^c^	[19]
Lifespan	Tumors do not reduce lifespan	Tumors reduce lifespan	Decreased survival rates in the mid- and high-dose groups ^d^	[76]

^a^ The highest tumor incidence after very low dose administration in the NTP series. Cancer in 39% of rats at very low doses; ^b^ Larger size of carcinomas show increasing metastatic capacity. Most non-metastatic carcinomas were <1.5 cm in diameter; ^c^ Unusually high incidence of metastases with 20% and 36% in the mid- and high-dose males with renal cancer, respectively; ^d^ 23/50 in the high dose surviving to terminal sacrifice compared with 39/50 in the controls.

## Abbreviations

The following abbreviations are used in this manuscript:

AIC: Akaike information criterion
AOAC: Association of analytical communities
BEN: Balkan Endemic Nephropathy
BMD: Benchmark dose
BMDL: Benchmark dose lower limit
BMR: Benchmark response
EDI: Estimated daily intake
EFSA: European food safety authority
GOF: Goodness-of-fit
ILSI: International life sciences institute
LOAEL: Lowest observed adverse effect level
LOD: Limit of detection
MOA: Mode of action
MOE: Margin of exposure
NCRI: Negligible cancer risk intake
NOAEL: No observed adverse effect level
NTP: National toxicology program
OTA: Ochratoxin A
PoD: Point of departure
SPE: Solid-phase extraction
TD: Tumorigenic dose
TDI: Tolerable daily intake
UF: Uncertainty factor
UTT: Urinary tract tumor
